# A tale of two curricula: Learning and matching in the final year of medical school

**DOI:** 10.36834/cmej.69239

**Published:** 2020-07-15

**Authors:** Lorenzo Madrazo

**Affiliations:** 1 University of Western Ontario, Ontario, Canada

## Abstract

In this article, I highlight two curricula that I believe are most prominent during the final year of the Canadian medical school experience—that of learning and that of matching to residency. While these two curricula are not mutually exclusive, they can be perceived as conflicting by learners who shift their focus away from learning towards performing in an effort to optimize their chance of matching to their program of choice. Moreover, the higher rates of unmatched students in recent years have likely contributed to this shift while at the same time bringing more stress and anxiety into the lives of medical students. I argue that there needs to be curricular consistency among all stakeholders including undergraduate programs, postgraduate programs, and other third-party organizations.

## Abstract (fr)

Dans cet article, je mets en évidence deux curriculums qui, je crois, prévalent durant la dernière année d’étude dans les écoles de médecine au Canada, soit celui de l’apprentissage et celui du jumelage aux postes de résidence. Alors que ces deux cursus ne sont pas mutuellement exclusif ils peuvent être perçus comme étant contradictoires par les apprenants qui, d’abord concentrés sur leurs apprentissages, priorisent ensuite la performance afin d’optimiser leur chance d’être jumelé au programme de leur choix. De plus, la proportion élevée d’étudiants non jumelés des dernières années a probablement contribué à ce changement de priorités infligeant, du même coup, plus de stress et d’anxiété dans leur vie d’étudiants en médecine. J’estime que le cursus soit être cohérent entre toutes les parties prenantes, incluant les programmes prédoctoraux, postdoctoraux, et les organisations de tierce partie.

“It was the best of times; it was the worst of times.”

From the frenzy of catching flights across the country, to the excitement of practicing medicine at different centres, to meeting application deadlines, to Match Day, writing the Medical Council of Canada Qualifying Examination (MCCQE) part 1, and graduation, the famous opening of *A Tale of Two Cities*,^1^ would aptly describe the range of emotions my colleagues and I experienced during our final year of medical school.

Reflecting on my final year, the title of Dickens’s book prompted a second comparison—that as learners, we receive two curricula or two “tales” that outline what our final year of medical school should be like and what its *telos* or purpose should be. On the one hand, students are told by administrators and leaders in medical education that our primary role is to be learners. Still officially undifferentiated, we are encouraged to seek out diverse clinical experiences in order to be well-rounded physicians.^2^ The increasingly interdisciplinary nature of Canadian medicine favours having a strong foundational knowledge in all aspects of medicine. On the other hand, there is a “not-so-hidden curriculum” (one well known to and understood by the students but hidden or ignored by the faculty) that preoccupies us with applying to our residency programs of choice, acquiring reference letters, and preparing our *curricula vitae* (CV) in the Canadian Residency Matching Service (CaRMS). Moreover, we are told explicitly by various academic advisors that we need to be *competitive*^3^ in order to stand out and match well to a desirable program.

Matching to your program of choice and learning are by no means incompatible. There is certainly much learning gained while trying to match well and likewise, aiming to be a well-rounded physician likely contributes to a successful match. Notwithstanding, problems arise when these messages are inconsistent and perceived by learners as contradictory to and conflicting with one another. The lack of clarity on the specific learning objectives of the final year of medical school remains an ongoing issue.^2^ Sometimes the poor design of the final year creates a learning environment that many students perceive as irrelevant or even as an impediment to matching well. Increasing the uncertainty and stress related to the matching process is the vague and unclear characterization of the ideal candidate, leading prospective residents to rely on unsubstantiated anecdotes and “rampant mythology.”^4^ It is no wonder, then, that final year medical students prioritize residency selection and preparation despite acknowledging the value of a broad learning experience.^5^^,^^6^ Moreover, a focus on matching well can cause learners to be disengaged from mandated learning activities or core rotations—what some call the “pre-residency syndrome.”^7^

Clinical electives are one area where the contradictions and conflicts between these two curricula are concretely manifested. From some school administrators, students are told that their objective as they approach electives is *learning* and exploring various areas of interest. They are encouraged to organize electives outside their desired specialty. On the other hand, students are advised by other mentors, residency program leaders, and senior peers to secure “audition electives”^8^ to survey a program of interest, make connections, and obtain a reference letter from that site to increase the chance of being selected by that program. While audition electives may be seen as necessary for some competitive specialties,^5^^,^^9^ I found no evidence to suggest that these practices, in general, substantially increase match rate success.^9^^,^^10^^,^^11^ Medical students can therefore be caught between choosing elective experiences that genuinely interest them and those they perceive will be beneficial for their residency applications but are not. More recently, the Association of Faculties of Medicine of Canada (AFMC) mandated a cap on the number of weeks in one specialty to promote the diversification of learning experiences and support reasonable match strategies.^12^ However, without similar changes in the postgraduate level that favour a diversity of electives (both formally and informally), medical students may continue to feel disadvantaged or even penalized for having a genuine interest in several specialties. Postgraduate medical education committees may perceive them as lacking interest or dedication to any particular specialty—even though taking a wide selection of electives is theoretically “the right thing to do.” Unfortunately, the higher rate of unmatched medical graduates in recent years has likely contributed to an increased focus on matching well and less focus on curricular obligations.^13^ The growing average number of residency applications^14^ likely reflects a rising anxiety about securing a desirable residency position.

Repeatedly, proposals have been put forward over the years calling for a more structured final year curriculum that provides a balance of broad learning experience as well as adequate preparation for residency.^15^^,^^16^ Curricular changes as well as policy changes like those mandated by undergraduate programs and the AFMC, respectively, are likely to be positive steps. Nevertheless, their effect is limited when not done in conjunction with *cultural* change characterized by close collaboration amongst all stakeholders—including those at both undergraduate and postgraduate levels. Inconsistency among these groups runs the risk of pursuing curricular reform in vain and could merely initiate changes that do not fundamentally address this problem we have identified over the past few decades.^2^^,^^16^^,^^17^ Curricular changes must also account for the current climate of higher unmatched rates and provide students with adequate supports. More importantly, all stakeholders have a role in advocating for improvements to the current way we facilitate the selection to residency positions.

Pursuing a consistent final year curriculum and a more collaborative medical education system will help teachers and learners strike the balance between learning and matching well in this pivotal year of medical training. Not only might these improvements help select for better residents for our patients—they might also allow medical students to look forward to their final year with great expectations.
